# Sustained low efficiency dialysis using a single-pass batch system in acute kidney injury - a randomized interventional trial: the REnal Replacement Therapy Study in Intensive Care Unit PatiEnts

**DOI:** 10.1186/cc11445

**Published:** 2012-07-27

**Authors:** Vedat Schwenger, Markus A Weigand, Oskar Hoffmann, Ralf Dikow, Lars P Kihm, Jörg Seckinger, Nexhat Miftari, Matthias Schaier, Stefan Hofer, Caroline Haar, Peter P Nawroth, Martin Zeier, Eike Martin, Christian Morath

**Affiliations:** 1 Department of Nephrology, University of Heidelberg, Im Neuenheimer Feld 672, Heidelberg 69120, Germany; 2 Department of Anesthesiology, Justus-Liebig-University Giessen, Rudolf-Buchheim-Strasse 7, Giessen 35392, Germany; 3 Section of Medical Statistics, University of Applied Science, Wiesenstrasse 14, Giessen-Friedberg 35390, Germany; 4 Department of Anesthesiology, University of Heidelberg, Im Neuenheimer Feld 672, Heidelberg 69120, Germany; 5 Department of Endocrinology, University of Heidelberg, Im Neuenheimer Feld 672, Heidelberg 69120, Germany

## Abstract

**Introduction:**

Acute kidney injury (AKI) is associated with a high mortality of up to 60%. The mode of renal replacement therapy (intermittent versus continuous) has no impact on patient survival. Sustained low efficiency dialysis using a single-pass batch dialysis system (SLED-BD) has recently been introduced for the treatment of dialysis-dependent AKI. To date, however, only limited evidence is available in the comparison of SLED-BD versus continuous veno-venous hemofiltration (CVVH) in intensive care unit (ICU) patients with AKI.

**Methods:**

Prospective, randomized, interventional, clinical study at a surgical intensive care unit of a university hospital. Between 1 April 2006 and 31 January 2009, 232 AKI patients who underwent renal replacement therapy (RRT) were randomized in the study. Follow-up was assessed until 30 August 2009. Patients were either assigned to 12-h SLED-BD or to 24-h predilutional CVVH. Both therapies were performed at a blood flow of 100 to 120 ml/min.

**Results:**

115 patients were treated with SLED-BD (total number of treatments n = 817) and 117 patients with CVVH (total number of treatments n = 877).The primary outcome measure, 90-day mortality, was similar between groups (SLED: 49.6% vs. CVVH: 55.6%, *P *= 0.43). Hemodynamic stability did not differ between SLED-BD and CVVH, whereas patients in the SLED-BD group had significantly fewer days of mechanical ventilation (17.7 ± 19.4 vs. 20.9 ± 19.8, *P *= 0.047) and fewer days in the ICU (19.6 ± 20.1 vs. 23.7 ± 21.9, *P *= 0.04). Patients treated with SLED needed fewer blood transfusions (1,375 ± 2,573 ml vs. 1,976 ± 3,316 ml, *P *= 0.02) and had a substantial reduction in nursing time spent for renal replacement therapy (*P *< 0.001) resulting in lower costs.

**Conclusions:**

SLED-BD was associated with reduced nursing time and lower costs compared to CVVH at similar outcomes. In the light of limited health care resources, SLED-BD offers an attractive alternative for the treatment of AKI in ICU patients.

**Trial registration:**

ClinicalTrials.gov NCT00322530

## Introduction

About 5 to 10% of patients admitted to the ICU develop acute kidney injury (AKI) requiring renal replacement therapy (RRT) [1]. AKI is an independent risk factor for mortality [1-3] and independently associated with the development of end-stage renal disease [4,5]. AKI is therefore not only one of the most important complications, but also one of the most cost-intensive interventions in ICU patients.

Recent randomized clinical trials showed no survival benefit or improved renal recovery in critically ill patients with AKI depending on the RRT, that is continuous RRT (CRRT) vs. intermittent renal replacement therapy (IRRT) [6-8], or the delivered dose of RRT [9-11]. More recently, newer hybrid techniques such as sustained low efficiency dialysis using a single-pass batch dialysis system (SLED-BD) which combines several advantages of both CRRT and IRRT have been introduced into clinical practice [12,13]. However, only small randomized controlled trials are available that compare SLED-BD with CRRT methods. Therefore, our study aimed to investigate survival, hemodynamic stability, practicability and costs in critically ill patients with AKI treated with either SLED-BD or continuous veno-venous hemofiltration (CVVH).

## Materials and methods

### Study settings

The renal replacement therapy study in ICU patients (RESCUE) is a prospective, randomized intention-to-treat study, conducted to compare two different RRTs in critically ill patients with AKI. The study was performed between 1 April 2006 and 31 January 2009 in the surgical ICU of the University of Heidelberg, Germany. The follow-up phase continued until 30 August 2009.

The study was registered [14] and the study protocol was approved by the local ethics committee (approval number 357/2005) and performed in accordance with the declaration of Helsinki and German Federal Guidelines. The integrity of data collection was independently verified by the Section of Medical Statistics, Technische Hochschule Mittelhessen (University of Applied Sciences), Germany. No manufacturer of dialysis devices was involved in the study design, data analysis or manuscript preparation.

### Study population

All patients admitted to the surgical ICU were screened for eligibility. Critically ill patients with AKI requiring RRT were eligible for enrolment if they were 18 years of age or older and met at least one of the following inclusion criteria: oligoanuria (urine output < 500 ml in a 24-hour period) and exclusion of post renal AKI, volume overload and unresponsiveness to fluid resuscitation measures, serum potassium > 6.5 mmol/l and an acute rise in plasma urea nitrogen level above 70 mg/dl.

Written informed consent was obtained from the patient or legal health care representative. Exclusion criteria were end-stage renal disease, preexisting chronic kidney disease stage 4 to 5 according to the Kidney Disease Outcome Quality Initiative (KDOQI), participation in another study, pregnancy and consent denial. Patients who had previously received renal replacement therapy during the same admission were ineligible for inclusion.

### Randomization and treatment assignments

Patients were randomly assigned to two treatment groups in a ratio of 1:1 using a computer-generated randomization modus. Patients allocated to the SLED-BD treatment (Genius®, Fresenius Medical Care, Bad Homburg, Germany) were assigned to receive 12-h of dialysis with a blood flow rate of 100 to 120 ml/min. For all SLED treatments, high-flux polysulfone filters (FX 50, Fresenius Medical Care, Bad Homburg, Germany) were used. In the Genius® dialysis device the dialysate is stored in an air-free 90-liter glass container (batch system) [15-17] (Additional file 1).

Patients randomly assigned to the CVVH-group (Prisma, Gambro Hospal, Lyon, France) were treated with 35 ml/kg per hour replacement fluid in predilution. Treatment was scheduled for 24-h and blood flow was maintained between 100 and 120 ml/min. For all CVVH treatments, high-flux polysulfone filters (Asahi KASEI APS-650, Asahi Kasei Medical Co, Ltd., Japan) were used.

It was in the responsibility of the ICU physician to decide whether to prolong or shorten the duration of treatment and whether to give blood transfusions; however, transfusion of packed red blood cells was recommended if the hemoglobin concentration fell below 7.0 g/dl [18].

### Study outcomes

The primary outcome measure of patients with AKI in the ICU was 90-day mortality. Secondary outcome measures included in-hospital mortality, mortality assessed until 30 August 2009, hemodynamic stability, time taken to recovery of renal function, duration of mechanical ventilation and length of ICU stay. A hypotensive episode was defined as an acute drop of systolic blood pressure below 80 mmHg or > 20% from baseline. The time to recovery of renal function was defined as the time from first RRT to last RRT and continuation of medical therapy. Recovery of renal function was defined as increasing diuresis without further need for RRT, stable or decreasing serum creatinine without RRT. Cessation of RRT was the responsibility of the ICU physician. Additional endpoints were treatment time of RRT (assessed at each day from 12 pm to 12 pm), number of packed red cell transfusions, nursing time spent for RRT and costs. Nursing time directly spent for RRT was documented at each RRT device contact. For the economic evaluation of the renal replacement modality, we documented the costs of nursing spent for each RRT, the costs of consumed RRT supplies and equipment, and the reimbursement by the German DRG (German diagnosis-related groups) system. All cost data were reported in euros.

### Statistical analysis

All analyses were performed according to the intention-to-treat principle. Probabilities of survival were calculated by the Kaplan Meier estimate and compared by application of the log-rank or Mantel-Haenszel test.

Comparison of qualitative data was performed using 2 × 2 contingency tables and Chi square analysis, with or without Yates' correction or by Fisher's exact test. For quantitative analysis, differences between means were identified using the Wilcoxon rank test for independent variables. Statistical significance was accepted at *P *< 0.05. Data in tables are presented as mean ± standard deviation.

All statistical analyses were performed using PCS software (PC statistics 5.0, O. Hoffmann, Giessen, Germany).

## Results

### Study enrollment and demographics

**Figure 1:**
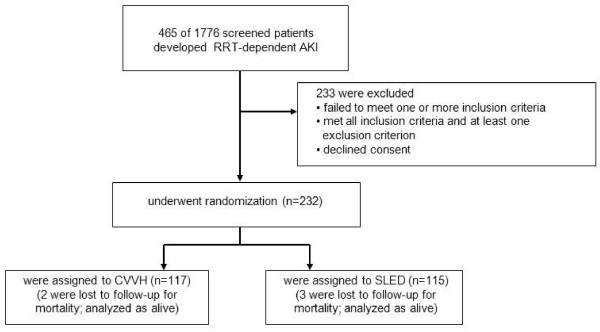
**Enrolment, randomization and inclusion of study patients**. Numbers of ICU patients enrolled in the study and randomly assigned to different treatment groups.

Between 1 April 2006 and 30 January 2009, 1,776 patients admitted to the surgical ICU were screened for eligibility; of these, 465 received RRT, 233 declined consent or failed the inclusion criteria and 232 were randomly assigned to one of the two treatment cohorts, SLED-BD: n = 115 vs. CVVH: n = 117 (Figure 1). A total of 15 randomized patients had an accidental change of the treatment modality in the daily routine. In detail, eight patients were randomized for CVVH but changed to SLED-BD, two were randomized to SLED-BD and changed to CVVH; three patients were randomized to SLED-BD and two to CVVH, but died before receiving any RRT.

As shown in Table 1, baseline characteristics and comorbidities were similar in both groups. Sepsis was the leading cause of AKI. Patients were comparable with respect to the mean Acute Physiology and Chronic Health Evaluation II score (APACHE II), the mean Therapeutic Intervention Scoring System (TISS) and the Simplified Acute Physiology Score II (SAPS II).

**Table 1:** Baseline characteristics of study patients.

	All (n = 232)	SLED (n = 115)	CVVH (n = 117)	*P*
Sex (female)	75 (32.3%)	43 (37.4%)	32 (27.4%)	0.135*
Age (years)	66.2 ± 12.4	66.6 ± 12.6	65.8 ± 12.1	0.252**
Body weight (kg)	80.4 ± 17.5	80.7 ± 19.2	80.2 ± 15.6	0.407**
Body mass index (kg/m^2^)	27.2 ± 5.2	27.5 ± 5.6	26.9 ± 4.7	0.417**
Diabetes prevalence	54 (23.4%)	25 (21.7%)	29 (25.0%)	0.667*
Coronary artery disease	75 (33.2 %)	39 (35.5%)	36 (31.0%)	0.573*
Cancer	91 (39.6%)	45 (39.1%)	46 (40.0%)	0.999*
Liver transplantation	46 (19.9%)	21 (18.3%)	25 (21.6 %)	0.644*
Kidney transplantation	2 (0.9%)	0	2 (1.7%)	0.481*
Serum creatinine (mg/dl)	2.57 ± 1.17	2.59 ± 1.33	2.55 ± 1.00	0.319**
Urea (mg/dl)	135.1 ± 56.7	132.7 ± 59.7	137.3 ±53.9	0.203**
Urine output (ml/24h)	2713 ± 2022	2786 ± 1970	2643 ± 2 081	0.275**
Oliguria (urine output < 500 ml/24h)	21 (14.0%)	9 (12.3%)	12 (15.6%)	0.368***
SAPS	68.5 ± 15.5	69.5 ± 14.0	67.6 ± 16.7	0.353**
Apache	31.8 ± 8.2	31.3 ± 8.7	32.2 ± 7.8	0.315**
TISS	48.7 ± 6.2	49.0 ± 5.4	48.5 ± 6.9	0.201**
Sepsis	121 (54.0%)	60 (54.0%)	61 (54.1%)	0.902*

All values are expressed as number (percent) or mean ± SD; statistical analyses were performed for SLED versus CVVH. *2 × 2 table Chi square test with Yates' correction; **one-tailed Wilcoxon test. *** Fisher's exact test. Apache: Acute Physiology and Chronic Health Evaluation II score; TISS: Therapeutic Intervention Scoring System; SAPS II: Simplified Acute Physiology Score II; SLED: sustained low efficiency dialysis; CVVH: continuous veno-venous hemofiltration.

### Treatment parameters and study outcomes

**Figure 2:**
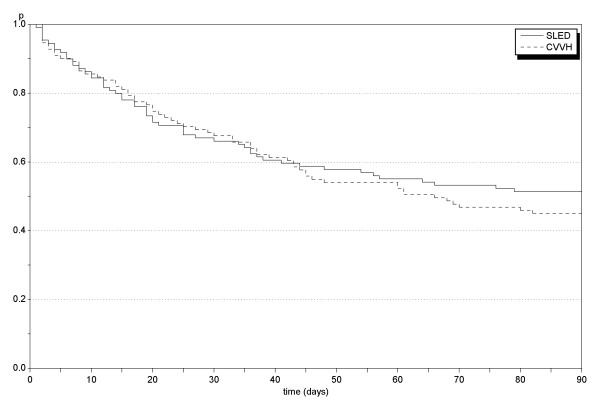
**Probabilities of survival in sustained low efficiency dialysis (SLED) and continuous veno-venous hemofiltration (CVVH) treatment groups (Kaplan-Meier estimates) during the first 90 days**. Mortality at 90 days was similar in ICU patients with acute kidney disease (AKI) treated with SLED-BD or with CVVH.

Treatment and follow-up data are presented in Tables 2 and 3. The primary outcome measure, 90-day mortality, as well as ICU mortality, in-hospital mortality, and long-term mortality (as assessed by August 30, 2009) were high in the study population but did not differ between the SLED and the CVVH groups (Table 3). Figure 2 gives the Kaplan-Meier survival estimates for both groups. Pre- and post-treatment systolic and diastolic blood pressure values and vasopressors were not significantly different; however, post-treatment systolic blood pressure tended to be higher in the SLED cohort (*P *= 0.05) (Table 3).

**Table 2:** Parameters and laboratory results during renal replacement therapy (RRT).

	All (n = 232)	SLED (n = 115)	CVVH (n = 117)	P*
Norepinephrine (µ/kg/min)	0.128 ± 0.129	0.137 ± 0.149	0.121 ± 0.11	0.266
Epinephrine (µ/kg/min) (n = 30)	0.100 ± 0.359	0.156 ± 0.233	0.243 ± 0.456	0.273
Dobutamine (µ/kg/min) (n = 109)	2.561 ± 1.446	2.710 ± 1.288	2.425 ± 1.576	0.015
Furosemide dose (mg/24h)	298.4 ± 239.5	311.0 ± 259.2	286.1 ± 218.9	0.365
Urine output (ml/24h)	1945 ± 1647	1860 ± 1693	2027 ± 1605	0.121
Duration of treatment (h) (values after exclusion of incomplete first and last treatment, see Methods)	16.7 ± 4.8	14.9 ± 4.4	15.9 ± 4.2 (19.9 ± 3.64)	0.024
Average number of treatments	7.67 ± 8.39	7.50 ± 8.89	7.83 ± 7.92	0.096
Absolute number of treatments	1694	817	877	
Net ultrafiltration (ml/24h)	1730 ± 1099	1850 ± 1179	1617 ± 1008	0.089
Fluid balance (ml/24h)	636 ± 1405	533 ± 1364	736 ± 1443	0.195
Effective blood flow (ml/min)	113.2 ± 15.5	125.7 ± 9.65	101.7 ± 9.82	< 0.001
Effluent flow (ml/h)			2390 ± 426	
Effluent flow rate (ml/kg/h)			30.87 ± 7.66	
Dialysate flow (ml/h)		120.9 ± 20.3		
Body temperature before RRT (°C)	36.5 ± 0.7	36.6 ± 0.7	36.4 ± 0.6	0.002
Change in body temperature during RRT (°C)	-0.2 ± 0.7	-0.3 ± 0.7	- 0.17 ± 0.7	0.011
Heparin (IU/24h)	4874 ± 4004	4554 ± 3594	5191 ± 4438	0.200
Hemoglobin (g/dl)	9.01 ± 0.95	9.06 ± 0.92	8.97 ± 0.97	0.276
Hematocrit (%)	0.29 ± 0.32	0.27 ± 0.03	0.30 ± 0.45	0.237
White blood cell count (/nl)	13.4 ± 7.3	13.0 ± 6.3	13.7 ± 8.2	0.460
Thrombocytes (/nl)	157.8 ± 123.5	157.2 ± 133.5	158.5 ± 113.4	0.177
C-reactive protein (mg/l)	107.5 ± 63.1	110.2 ± 66.8	105.1 ± 59.4	0.392
Albumin (g/l)	23.9 ± 4.6	24.1 ± 4.6	23.7 ± 4.6	0.306
pH	7.38 ± 0.05	7.38 ± 0.06	7.38 ± 0.05	0.242
HCO3- (mmol/l)	23.6 ± 2.55	23.6 ± 2.47	23.6 ± 2.62	0.478
Lactate (mg/dl)	21.1 ± 25.3	19.4 ± 22.9	22.7 ± 27.5	0.260
Serum creatinine pre-treatment (mg/dl)	1.85 ± 0.80	1.77 ± 0.90	1.92 ± 0.68	0.007
Serum creatinine after treatment (mg/dl)	1.46 ± 0.75	1.26 ± 0.80	1.66 ± 0.65	< 0.001
Urea pre-treatment (mg/dl)	113.5 ± 48.3	105.7 ± 55.7	121.2 ± 38.4	0.002
Urea after treatment (mg/dl)	87.1 ± 35.6	68.4 ± 31.2	105.5 ± 29.7	< 0.001
Phosphate pre-treatment (mmol/l)	1.39 ± 1.50	1.42 ± 2.03	1.35 ± 0.52	0.018
Phosphate after treatment (mmol/l)	1.02 ± 0.37	0.88 ± 0.34	1.18 ± 0.35	< 0.001
Calcium (mmol/l)	2.07 ± 0.19	2.04 ± 0.16	2.10 ± 0.22	0.007
Total packed red blood cell Transfusion per patient (ml)	1680 ± 2981	1375 ± 2573	1976 ± 3316	0.019
Total fresh frozen plasma per patient (ml)	1586 ± 4290	1285 ± 3942	1878 ± 4603	0.115

All values are expressed as daily mean (unless otherwise indicated) ± SD; statistical analyses were performed for SLED versus CVVH; *one-tailed Wilcoxon test. SLED: sustained low efficiency dialysis; CVVH: continuous veno-venous hemofiltration.

**Table 3:** Primary and secondary outcomes.

	All (n = 232)	SLED (n = 115)	CVVH (n = 117)	*P*
Death from any cause by day 90	122 (52.6 %)	57 (49.6 %)	65 (55.6 %)	0.434**
Death from any cause up to 30 August 2009	155 (66.8 %)	76 (66.1 %)	79 (67.5 %)	0.926**
In-hospital mortality	119 (51.3 %)	57 (49.6 %)	62 (53.0 %)	0.696**
Mortality in ICU	98 (42.2 %)	49 (42.6 %)	49 (41.9 %)	0.984**
Mechanical ventilation	205 (88.4%)	101 (87.8%)	104 (88.9%)	0.962**
Days of mechanical ventilation	1 9.4 ± 19.7	17.7 ± 19.4	20.9 ± 19.8	0.047*
Days in intensive care unit	21.7 ± 21.1	19.6 ± 20.1	23.7 ± 21.9	0.038*
Recovery of kidney function in days after RRT initiation	10.2 ± 14.5	10.0 ± 15.2	10.5 ± 14.0	0.049*
BP syst pre-treatment (mmHg)	124.8 ± 14.0	125.1 ± 14.6	124.6 ± 13.5	0.434*
BP syst after treatment (mmHg)	126.3 ± 16.4	128.3 ± 17.1	124.3 ± 15.6	0.051*
BP diast pre-treatment (mmHg)	60.7 ± 10.3	60.7 ± 10.7	60.7 ± 10.0	0.420*
BP diast after treatment (mmHg)	61.1 ± 10.7	61.8 ± 11.3	60.3 ± 10.2	0.250*
Hypotensive episodes	1.6 ± 1.5	1.5 ± 1.4	1.8 ± 1.6	0.077*

All values are expressed as number (percent) or mean ± SD; statistical analyses were performed for SLED versus CVVH. *One-tailed Wilcoxon test; **2 × 2 table Chi-Square test with Yates' correction. BP syst: systolic blood pressure; BP diast: diastolic blood pressure; SLED: sustained low efficiency dialysis; CVVH: continuous veno-venous hemofiltration.

SLED was accomplished with a significantly higher effective blood flow (*P *< 0.001), whereas ultrafiltration (UF) volume (*P *= 0.08), the fluid balance and the number of hypotensive episodes did not differ between groups (Tables 2 and 3). The decrease in body temperature during treatment was more pronounced in the SLED group (Table 2) and was negatively correlated with change in diastolic but not systolic blood pressure (r = -0.175, *P *= 0.008) (Table 2).

Duration of mechanical ventilation (*P *= 0.047) and of ICU-stay (*P *= 0.038), and time to renal recovery (*P *= 0.049) were significantly shorter in the SLED group (Table 3). The average number of treatments was similar in both groups (*P *= 0.096). Although a 12-h treatment period for SLED, and a 24-h treatment period for CVVH was anticipated, the average of the delivered treatment time differed only by 1 h (*P *= 0.024). If the incomplete first and last treatments (due to the assessment from 12.00 pm to 12.00 pm, see methods) were excluded, the average treatment time for CVVH was 19.9 ± 3.64 h (median 20.8 h) (Table 2). The delivered CVVH dose was below the prescribed dose at 31 ml/kg per h, but within the recommended dose for CVVH treatment [19]. Solute clearance (as demonstrated by urea and phosphate values) was more effective in the SLED-treated patients than in the CVVH-treated patients (Table 2).

The number of dialysis catheters used per treatment did not differ between both groups. However, the number of membranes used per patient was higher in the SLED group (Table 4). The quantity of blood transfusions given was significantly higher in the CVVH than in the SLED patient cohort, although the mean daily heparin dose did not differ (Table 2). No difference was observed in the administration of fresh frozen plasma.

**Table 4:** Dialysis equipment and nursing time per patient.

	SLED (n = 115)	CVVH (n = 117)	P*
Number of membranes used	14.3 ± 15.3	7.56 ± 8.54	< 0.001
Number of dialysis catheters used	1.74 ± 1.44	1.70 ± 1.89	0.294
Nursing time spent (minutes)			
< 1	9.36 ± 14.7	14.3 ± 41.9	0.131
1 to 5	3.64 ± 4.63	6.75 ± 13.2	0.136
> 5	7.24 ± 9.51	12.1 ± 17.0	< 0.001

*One-tailed Wilcoxon test. SLED: sustained low efficiency dialysis; CVVH: continuous veno-venous hemofiltration.

### Economics

The nursing time spent for RRT was significantly higher in the CVVH group than in the SLED group (Table 4). Evaluating the expenditure and lower direct costs for providing RRT, SLED used with a specific AKI membrane was associated with lower costs of about €220 per patient compared with CVVH (Tables 4 and 5). If conventional high-flux membranes were used instead of specific AKI membranes (as was performed in our study), the cost difference increased to €1,300 per patient (the acquisition costs for the Genius® facility were considered with an advance payment of €8 per treatment). SLED, using a conventional high-flux membrane, was associated with significantly lower costs even after consideration of the higher reimbursement for CVVH by the German DRG system (Table 5).

**Table 5:** Estimated costs of renal replacement therapy (RRT).

	SLED (using a high-flux membrane)	SLED (using an AKI membrane)	CVVH
RRT costs/day (acquisition cost for the Genius and water preparation device included with €8/treatment. Number of membranes used per treatment were considered)	€63.2	€206.7	€209.3
			
Overall modality costs per treatment (nursing included, without technician, physician and other medical staff)	€96.8	€240.4	€258.9
			
Reimbursement for modality/day (German DRG system)	€221.0	€221.0	€300.0

DRG: German diagnosis-related groups; SLED: sustained low efficiency dialysis; CVVH: continuous veno-venous hemofiltration.

## Discussion

In this prospective randomized study, we compared CVVH with a targeted dose of 35 ml/kg per h with SLED-BD for the treatment of AKI in ICU. In line with other reports on patients with sepsis and multi-organ failure, we found high 90-day mortality, and no differences in mortality at any time (for ICU-, in-hospital, or long-term mortality) [1,7,8].

Recent data demonstrate that neither the technique of RRT [7,8] nor the dose of RRT [9-11] had an impact on patient survival. In the light of the markedly higher costs of CRRT, it was therefore suggested that, in the absence of a survival benefit of CRRT, intermittent hemodialysis (IHD) should be the preferred treatment modality for AKI in critically ill patients [20]. In the meantime, the newer hybrid technique, SLED, which combines the hemodynamic stability of CRRT with a more favorably priced dialysis technique, has been introduced as a new cost-effective approach to the treatment of AKI in the ICU [13,21,22]. The hemodynamic stability of this approach was demonstrated in the first prospective smaller investigations [12,21]. This study is, to the best of our knowledge, the first larger randomized study comparing SLED-BD with CVVH. As demonstrated, blood pressure was well maintained and even tended to be higher after treatment in the SLED group compared to CVVH, although the net UF and the use of vasopressors were not significantly different with either therapy. The decreased temperature of the non-heated dialysate during SLED-BD might have accounted for this; and in fact, the decrease in body temperature after treatment in the SLED-BD patient cohort was greater than in the CVVH treated cohort. However, the maintained hemodynamic stability in the SLED treated group may also be the result of the extended duration of the SLED treatment prescribed by the ICU physician in charge. Primarily SLED was prescribed to deliver a treatment time of 12 hours; however, the actual delivered treatment time was prolonged by the ICU physician to 14.9 hours. In contrast, the actual treatment time in the CVVH cohort was reduced but was still significantly higher than the treatment time for SLED. Although the average treatment time seems rather low, we assume that the actual delivered treatment time for CVVH in this study reflects clinical practice, at least in surgical units, where treatment is often interrupted due to bleeding complications, operative revisions or consecutive investigations. In addition, one has to consider, that we evaluated the treatment time from 12 pm to 12 pm, which means that if a treatment starts in the afternoon of the first day at 6 pm, the first session was documented as a 6 h treatment (from start until midnight). This applies also for the last treatment. When we excluded the incomplete first and last CVVH treatments, the mean duration of treatment was 19.9 ± 3.64 hours. Although, this is in line with current practice [10], this emphasizes that the method of time evaluation of treatment is of great importance and should be given. It is of interest that in almost all studies on CRRT, the prescribed and delivered doses are mentioned, but the treatment time per day and the time of interruption of treatment are not [7-9,23]. This is of importance, as some studies demonstrate that the time of interruption of CRRT might be relevant [11].

Our data illustrate that SLED treatment was adapted by the intensivists to a CRRT modality. In the face of newer hybrid techniques the classical differentiation in CRRT (mostly performed by CVVH with convective elimination of solutes) and IRRT (hemodialysis with diffusive elimination of solutes) may become obsolete. One has to keep in mind that adapting the treatment time of a dialysis device to a CRRT modality, resulted not only in a higher solute clearance but might lead to a potentially dangerous underdosing of, for example, anti-infective agents [24].

Another aspect, apart from the actual delivered treatment time, is that it still remains a matter of debate when to start, but also when to stop RRT, and this discussion is ongoing. It might be considered that the start of RRT in our study was too early (or too late), however, even today, criteria for starting and ending RRT remain arbitrary in many respects.

A potentially complicating factor is the more intensified anticoagulation for CRRT due to the longer treatment time required to prevent clotting of the extracorporeal circuit. In fact, earlier reports have demonstrated a significantly lower need for anticoagulation in SLED-treated patients [12,21,25]. However, the total amount of heparin administered in our study was slightly, but not statistically significantly lower in patients treated with SLED. This clearly overcomes the prejudice that CRRT *per se *requires a more intensified anticoagulation; the need for anticoagulation seems rather a consequence of the treatment time than a consequence of the treatment modality used. We do not believe that a slightly but insignificantly increased amount of heparin infused in CVVH patients in this study resulted in a higher quantity of transfused red packed cells. We may eventually only speculate as to whether small but insignificant differences in volume balance might be causative for the observed differences in blood transfusions or in the number of days of mechanical ventilation. On the other hand, the longer duration of mechanical ventilation might be responsible for the slight difference observed in the time for recovery of kidney function [26].

Costs for medical devices and treatment are of increasing importance. CVVH treatment in general consumes greater health care resources than dialysis techniques [20]. We evaluated the modality-associated costs of SLED, taking the acquisition costs for the dialysis facility into account, and compared them with CVVH for ICU patients with AKI, based on the average purchase price, number of membranes used, personnel costs and the reimbursement by the German DRG system. SLED was associated with a reduction in cost for providing RRT. In addition, a further cost reduction for the SLED modality was achieved in our department by substituting the specific AKI membrane by a conventional high-flux membrane. The costs for RRT (treatment and nursing costs) differed by about €220 per ICU patient. In fact, the difference would be much higher using conventional high-flux membranes rather than the specific AKI membranes used in our study. The difference was about €1,300 per patient. Further cost savings may be achieved by the use of a newer biocompatible low-flux membrane, which has almost equivalent efficacy to high-flux membranes at lower costs [27,28], or by the substitution of heparin anticoagulation by using regional citrate anticoagulation protocols [29]. Though a large number of membranes were used this is in line with other studies [9]. During cost calculations, one has also to consider that the hospital reimbursement by the German DRG system is markedly higher for CVVH compared to dialysis devices. Taking the lower direct costs of SLED and the higher reimbursement for CVVH into account, our data demonstrate that CVVH remains still more expensive than SLED without superior patient outcome, at least in this small patient cohort of surgical ICU patients. Therefore, using a SLED-BD system in AKI patients in ICU has distinct advantages with regard not only to cost effectiveness, but also patency and practicability.

### Limitations

There are several limitations of the study. First, the results of this study are from a small patient cohort from a surgical ICU and therefore may not be easily extrapolated to non-surgical ICU patients. Second, the power of the study may be insufficient to finally judge whether one or the other treatment modality is superior in terms of survival. However, the power is clearly sufficient to discriminate differences in economics; with a significance level of 0.05, the estimated power is 99% for the number of membranes used and 81% for more than 5 minutes nursing time spent directly on the device (data not shown in the results). Third, the timing of the start and end of RRT is still a matter of debate but might have influenced the results in this study. Finally, though the targeted treatment times for CVVH and SLED-BD differed by 12 hours, the difference of the delivered treatment time was much lower.

## Conclusions

Newer hybrid SLED techniques using a single-pass batch system demonstrated similar patient outcomes and hemodynamic properties compared to CVVH but were associated with a more favorable price. In the light of limited health care resources, SLED offers an attractive alternative for treatment of AKI in ICU patients.

## Key messages

• Mortality and hemodynamic stability did not differ in AKI patients treated either with SLED or CVVH

• SLED treatment was associated with a lower quantity of blood transfusions given, a shorter ICU stay, shorter duration of mechanical ventilation and faster recovery of renal function

• SLED was accompanied with reduced nursing time spent for RRT and lower costs for providing RRT

## Abbreviations

AKI: acute kidney injury; Apache II: Acute Physiology and Chronic Health Evaluation II score; CRRT: continuous renal replacement therapy; CVVH: continuous veno-venous hemofiltration; German DRG: German diagnosis-related groups; ICU: intensive care unit; IHD: intermittent hemodialysis; IRRT: intermittent renal replacement therapy; KDOQI: Kidney Disease Outcome Quality Initiative; RESCUE: renal replacement therapy study in ICU patients; RRT: renal replacement therapy; SLED: sustained low efficiency dialysis; SLED-BD: sustained low efficiency dialysis using a single-pass batch dialysis system; SAPS II: Simplified Acute Physiology Score II; TISS: Therapeutic Intervention Scoring System; UF: ultrafiltration; UV: ultraviolet.

## Competing interests

VS, LPK, CM, MAW received lecture fees from Fresenius Medical Care, Germany. VS received scientific grants from Gambro Corporation Research, Germany. The other authors have no conflict of interest with regards to the manufacture of the renal replacement therapy devices mentioned in the manuscript.

## Authors' contributions

VS, CM and MAW contributed to the study conception and design and drafted the manuscript. RD, LPK, JS, NM, MS, SH, CH, MAW performed RRT and contributed to patient recruitment. MZ, EM and PPN helped to draft the manuscript. OH performed the statistical analysis and survival figures. All authors have read and approved the final version of the manuscript.

## Supplementary Material

Additional file 1 **figure showing a single-pass batch dialysis device (with courtesy Fresenius Medical Care, Germany)**. Fresh dialysate is aspirated from the top of the tank via a roller pump, whereas spent dialysate is returned to the bottom of the glass tank. Recently, the nearly complete separation of spent and fresh dialysate was demonstrated in detailed studies [15,30]. (1) Double-sided blood as well as dialysis pump. (2) Air detector between pump and membrane. (3) Dialysis membrane. (4, 5) Air-free flow chamber. (6) Ultrafiltration pump (ultrafiltration is removed by volumetric control via a roller pump). (7) Ultrafiltration collecting-container. (8) Upper part of the tank (fresh dialysate is removed from here). (9) Water boundary layer from fresh dialysate in the upper part and spent dialysate in the lower part. (10) Spent dialysate. (11) UV-radiator. (12) Glass container with thermal insulation. Click here for file
