# Supplementary figures and images for: Sustained low efficiency dialysis using a single-pass batch system in acute kidney injury - a randomized interventional trial: the REnal Replacement Therapy Study in Intensive Care Unit PatiEnts
Source: Crit Care. 2012 Jul 27;16(4):R140. doi: 10.1186/cc11445 (PMC3580725; doi:10.1186/cc11445)

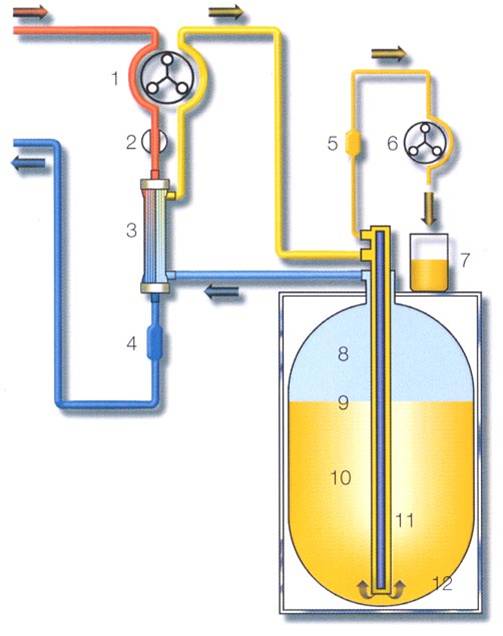

Supplement: Additional file 1 — figure showing a single-pass batch dialysis device (with courtesy Fresenius Medical Care, Germany). Fresh dialysate is aspirated from the top of the tank via a roller pump, whereas spent dialysate is returned to the bottom of the glass tank. Recently, the nearly complete separation of spent and fresh dialysate was demonstrated in detailed studies [15,30]. (1) Double-sided blood as well as dialysis pump. (2) Air detector between pump and membrane. (3) Dialysis membrane. (4, 5) Air-free flow chamber. (6) Ultrafiltration pump (ultrafiltration is removed by volumetric control via a roller pump). (7) Ultrafiltration collecting-container. (8) Upper part of the tank (fresh dialysate is removed from here). (9) Water boundary layer from fresh dialysate in the upper part and spent dialysate in the lower part. (10) Spent dialysate. (11) UV-radiator. (12) Glass container with thermal insulation. [file cc11445-S1.JPEG]
